# PLGA Nanospheres as Delivery Platforms for *Eimeria mitis* 1a Protein: A Novel Strategy to Improve Specific Immunity

**DOI:** 10.3389/fimmu.2022.901758

**Published:** 2022-05-26

**Authors:** Lixin Xu, Zhengqing Yu, Ke He, Zhaohai Wen, Muhammad Tahir Aleem, Ruofeng Yan, Xiaokai Song, Mingmin Lu, XiangRui Li

**Affiliations:** Ministry of Education (MOE) Joint International Research Laboratory of Animal Health and Food Safety, College of Veterinary Medicine, Nanjing Agricultural University, Nanjing, China

**Keywords:** *Eimeria mitis*, 1a protein, PLGA, chitosan, emulsion, protective efficacy

## Abstract

The infections of chicken coccidiosis impact the welfare of chickens and the economical production of poultry. *Eimeria mitis* is ubiquitous in chicken coccidiosis, and *E. mitis* infection can significantly affect the productivity of birds. Up to now, few efficient vaccines against *E. mitis* have been reported, whereas the recombinant subunit vaccines delivered by nanomaterials may elicit an encouraging outcome. Thus, in this study, we chose *E. mitis* 1a (Em1a) protein as the candidate antigen to generate Em1a preparations. The recombinant Em1a (rEm1a) protein was encapsulated with poly lactic-co-glycolic acid (PLGA) and chitosan (CS) nanospheres. The physical characterization of the rEm1a-PLGA and rEm1a-CS nanospheres was investigated, and the resulting nanospheres were proven to be nontoxic. The protective efficacy of rEm1a-PLGA and rEm1a-CS preparations was evaluated in *E. mitis*-challenged birds in comparison with two preparations containing rEm1a antigen emulsified in commercially available adjuvants. ELISA assay, flow cytometry analysis, and quantitative real-time PCR (qPCR) analysis indicated that vaccination with rEm1a-loaded nanospheres significantly upregulated the secretions of antibodies and cytokines and proportions of CD4^+^ and CD8^+^ T lymphocytes. Compared with the other three preparations, rEm1a-PLGA nanosphere was more effective in improving growth performance and inhibiting oocyst output in feces, indicating that the PLGA nanosphere was associated with optimal protection against *E. mitis*. Collectively, our results highlighted the advantages of nanovaccine in eliciting protective immunity and may provide a new perspective for developing effective vaccines against chicken coccidiosis.

## Introduction

As one of the major diseases in poultry production, chicken coccidiosis is mainly induced by single or multiple infections of the *Eimeria* species, leading to an annual economic loss of about 10.4 billion francs worldwide (1). Traditionally, seven *Eimeria* species with variable levels of pathogenicity, namely, *Eimeria acervulina*, *Eimeria brunetti*, *Eimeria necatrix*, *Eimeria tenella*, *Eimeria maxima*, *Eimeria mitis*, and *Eimeria praecox*, are considered to be infectious to the chicken (2). Once chickens ingest sporulated *Eimeria* oocysts, coccidiosis infection can cause nutrient absorption deficiency, poor growth performance, and, in some cases, significant mortality (3). Although *E. mitis* and *E. praecox* are usually considered as the less-pathogenic *Eimeria* species (4, 5), their transmissions in chickens can significantly reduce the productivity of hosts, posing an economic threat to the poultry industry (6, 7). Furthermore, numerous studies have revealed that *E. mitis* are associated with growth reductions and low egg productivity in laying hens (4, 6, 8–10). At present, anticoccidial drugs tend to become the predominant way to control chicken coccidiosis. However, the rise of antibiotic resistance and the limited use of chemical drugs in food animals have recently driven the rapid development of vaccines against coccidiosis (11, 12).

Currently, numerous vaccines were reported on the pathogenic *Eimeria* species, such as *E. tenella*, *E. acervalina*, *E. maxima*, and *E. necatrix* (13, 14). However, the vaccines against *E. mitis* did not cause wide public concern over the recent years, and the synergistic mechanisms after coinfection with *E. mitis* still remain unclear. Thus, efficient and safe vaccines against *E. mitis* may be an easy shortcut to reducing economic losses (15). Detailed research of the *Eimeria* life cycle has revealed many protein antigens that are recognized as potential candidates for vaccines (16, 17). Examples include apical membrane antigen (14), microneme (17, 18), surface antigen (19), immune mapped protein (16), and profilin (20). In addition, Liu et al. (21) reported on the potential vaccine candidates of 14-3-3 proteins with a conserved sequence in parasites. However, these reported vaccines cannot provide complete protection for chickens. As the research related to metabolism-related genes develops, more and more vaccine targets were discovered and exhibited satisfactory immunoprotection against other Apicomplexan parasites (22, 23). Being homologous with the nicotinamide-nucleotide-dependent transhydrogenase and described as the refractile body protein (24), 1a protein probably participated in carbohydrate transport for containing a sequence characterized by hexose transporters (24). Such a hypothesis is confirmed by the associations of the refractile bodies and amylopectin granules (25, 26), suggesting that 1a proteins play a critical role in metabolism and energy storage in *Eimeria* species. These reports indicated a vital role of 1a proteins in the survival of *Eimeria* species, and the construction of anti-*Eimeria* vaccines targeting the 1a proteins seems to be rational in eliciting protective efficacy against chicken coccidiosis.

Traditional vaccine strategies against *Eimeria* species mainly focused on the live, attenuated, inactivated, recombinant subunit, and DNA vaccines (13). Unfortunately, attenuated vaccines allowed the duplication of *Eimeria* species within animals, leading to the risk that *Eimeria* may revert to full virulence (27). Limitations also occurred in inactivated vaccines for their short duration of induced immune response (28). DNA vaccines can effectively avoid these problems, but the theoretical risk of exogenous gene integration into host genomes cannot be ignored (28). Among many types of vaccines, recombinant subunit vaccines gained our particular interest. By inserting mutagenesis and undesirable sequences, recombinant subunit vaccines can stop the reversal of toxoids back to full virulence (29, 30). However, recombinant subunit vaccines are easily biodegraded by enzymes, and an effective adjuvant is essential to prevent peptides from undesirable degradation and strengthen immunogenicity (31, 32). Recently, the nanomaterials that served as the nanospheres to load peptides have emerged as one of the most efficient strategies to induce robust immunity (33, 34). Approved by the Food and Drug Administration (FDA) and European Medicines Agency (EMA), poly lactic-co-glycolic acid (PLGA) is a synthetic polymer that has been widely used in various vaccines and drugs. Nanospheres formulated by PLGA have been proven to be efficient in peptide delivery due to their biocompatibility, nontoxicity, and biodegradability (35). While in comparison with the cationic polymers, PLGA nanospheres also exhibited certain disadvantages in peptide delivery. They will encounter instability and low encapsulation efficiency (EE) when loading negative molecules. In addition, with biocompatibility, relative safety, and biodegradability (36), chitosan (CS) has been widely used in various industrial areas including the food industry and biomedical materials (37, 38).

In this study, we chose the *E. mitis* 1a (Em1a) protein as the candidate antigen to construct a recombinant subunit vaccine against *E. mitis*. The recombinant Em1a protein (rEm1a) was encapsulated with PLGA or CS nanomaterials to generate Em1a-PLGA and Em1a-CS nanospheres. The protective efficacy of the resulting preparations was evaluated in *E. mitis*-challenged birds in comparison with the preparations containing rEm1a antigen emulsified in commercially available adjuvants. Our results highlighted the advantages of the newly constructed nanovaccine in eliciting robust immunity, and it could be used as an alternative strategy against *E. mitis* infection but with high priority.

## Materials and Methods

### Animals and Parasites

The newborn Hy-Line (breed W-36) chickens were obtained from Shuangli Hatchery, Nantong, China, and the animals were hatched in a coccidia-free condition. Free of vaccination, all chickens were raised in a coccidia-free environment and had free access to coccidiostat-free water and food. Purchased from the Model Animal Research Center, Nanjing University, Nanjing, China, specific pathogen-free (SPF) BALB/c mice (weighing 18–22 g) were fed in the SPF condition. Under the supervision of the Animal Ethics Committee of Nanjing Agriculture University, Nanjing, China, the operations associated with animals were conducted in strict compliance with the requests of the Ethics Procedures and Guidelines of the People’s Republic of China.

Purified *E. mitis* oocysts were provided by the Ministry of Education (MOE) Joint International Research Laboratory of Animal Health and Food Safety, College of Veterinary Medicine, Nanjing Agricultural University, Nanjing, China, and preserved in 2.5% potassium dichromate at 4°C. One week prior to the challenge, the *E. mitis* oocysts were propagated, accumulated, and sporulated according to a previous paper (39).

### Cloning and Plasmid Construction

The genomic DNA of sporulated oocysts was isolated by E.Z.N.A.^®^ Stool DNA Kit (Omega Bio-Tek, Norcross, GA, USA), and the molecular identification was conducted as described previously (40–42) to exclude the contamination of other *Eimeria* species. The primers used here are listed in **Table S1**. Total RNA of 10^7^ purified *E. mitis* oocysts was extracted by applying the TRIzol reagent (Vazyme Biotech Co., Ltd., Nanjing, China). Complementary DNA (cDNA) was synthesized using the reverse transcription kit (Vazyme Biotech Co., Ltd., Nanjing, China). Along with restriction endonuclease sites (*Kpn*I and *Eco*RV), primers were designed based on the conserved domain sequences (CDSs) of Em1a (GeneBank: HQ148300.1). The forward (5′-CGGGGTACCATGCCTCCCTCCGCTG-3′) and reverse (5′-GGATATCTTATCTTGAGACGGGCGTT-3′) primers were synthesized by Tsingke Biological Technology (Nanjing, China). According to the specification of High-Fidelity Master Mix (Tsingke Biological Technology), the PCR reaction was carried out in a volume of 50 μl under the following condition: 95°C for 5 min, followed by 35 cycles of 95°C for 30 s, 56.1°C for 30 s, and 72°C for 80 s; a final extension at 72°C for 5 min was also conducted. The amplicons were purified by Gel Extraction Kit (Omega Bio-Tek), digested by *Kpn*I and *Eco*RV restriction endonuclease (Takara Biotechnology, Dalian, China), and subcloned to a linearized pET-32a prokaryotic vector (Invitrogen Biotech, Carlsbad, CA, USA). Then, the recombinant vector (named pET-32a-Em1a) was transferred into the *Escherichia coli* DH5α chemical-competent cells (Tsingke Biological Technology) and propagated in Luria–Bertani (LB) medium containing 100 μg/ml ampicillin. The pET-32a-Em1a plasmid was extracted by a Plasmid Mini Kit (Omega Bio-Tek) and determined by double restriction enzyme digestion and PCR using the ABI PRISM™ 3730 XL DNA Analyzer (Applied Biosystems, Waltham, MA, USA). The correct plasmid was then transferred into *E. coli* BL21 (DE3) chemical-competent cells (Tsingke Biological Technology).

### Preparation of the Purified rEm1a Protein

To obtain the rEm1a protein, *E. coli* BL21 (DE3) chemical-competent cells carrying the recombinant pET-32a-Em1a plasmid were grown in the LB medium containing 100 μg/ml ampicillin at 37°C (180 rpm) until OD600 reached approximately 0.5. Then, the expression of rEm1a was induced by 1.0 mM isopropyl β-D-thiogalactoside (IPTG; Sigma, St. Louis, MO, USA). After 4 h of incubation, the bacterial cells were gathered by centrifugation at 8,000 rpm for 15 min at room temperature. The rEm1a was then purified by a HisTrap™ FF chelating column (Cytiva, Marlborough, MA, USA) according to the manufacturer’s guidelines. The ToxinEraser™ Endotoxin Removal Kit (GeneScript, Piscataway, NJ, USA) was used to remove the endotoxin from purified rEm1a protein. Then, the obtained proteins were analyzed by ToxinSensor™ Chromogenic LAL Endotoxin Assay Kit (GeneScript, Piscataway, NJ, USA) and 12% (*w*/*v*) sodium dodecyl sulfate-polyacrylamide gel electrophoresis (SDS-PAGE) for the determination of the endotoxin level and the purity of rEm1a proteins, respectively. Purified rEm1a protein was kept at -80°C until use. Before subsequent analysis, the concentrations of rEm1a were determined by the Pierce™ BCA Protein Assay Kit (Thermo Scientific, Waltham, MA, USA).

### Immunoblot Analysis of rEm1a Proteins

To obtain the serum against *E. mitis*, each coccidia-free chicken was raised in a coccidia-free environment. At the age of 14 days, birds were orally challenged with 5 × 10^4^ sporulated oocysts four times with an interval of 7 days. Before challenge, genotyping was conducted to determine whether the oocysts were contaminated by other *Eimeria* species. One week after the last infection, blood samples were harvested from the brachial wing vein of challenged birds. Meanwhile, control samples were also obtained from the phosphate-buffered saline (PBS)-infected chickens. The serum was then separated and stored at -20°C until use.

To investigate the recognition of rEm1a protein by chicken anti-*E. mitis* serum, purified rEm1a proteins were first resolved on 12% SDS-PAGE gel and subsequently transferred to polyvinylidene fluoride (PVDF) membranes (Millipore Ltd., Tullagreen, Co., Cork, Ireland) through a Trans-Blot Semi-Dry Transfer Cell System (Bio-Rad, Hercules, CA, USA). Then, the PVDF membranes were blocked in Tris-buffered saline (TBS) containing 5% (*w*/*v*) skimmed milk powder and 0.5% (*v*/*v*) Tween 20 at 37°C for 2 h. Rinsed in TBS containing 0.5% (*v*/*v*) Tween 20 (TBST) at room temperature for 5 min, PVDF membranes were incubated with chicken sera in blocking buffer (1:100 dilutions) at 4°C on a rotary shaker (30 rpm) overnight. Rinsed again in TBST at room temperature for 5 min, PVDF membranes were incubated with HRP-conjugated goat anti-rat IgG (eBioscience, San Diego, CA, USA) in blocking buffer (1:8,000 dilutions) at 37°C on a rotary shaker (30 rpm) for 2 h. Finally, PVDF membranes were rewashed, and bound antibodies were visualized by utilizing the electrochemiluminescence (ECL) system (Tanon, Shanghai, China).

### Vaccine Synthesis

To obtain the Em1a-71VR emulsions (water-in-oil emulsion), Montanide™ ISA-71R VG (Seppic, Paris, France) was emulsified with purified rEm1a proteins at a ratio of 7:3 under room temperature according to the instructions. For complete emulsion, the mixtures were stirred by a T10 blender (IKA, Staufen, German) at 15,600 rpm for at least 15 min until the homogeneous emulsion was formed. To avoid antigen degradation, the resulting Em1a-71VR emulsions were stored at 4°C for less than 2 h until use.

As for the Em1a-201VG emulsions (water-in-oil-in-water emulsion), Montanide™ ISA-201VG (Seppic) was first preheated to 50°C, and then the PBS-diluted rEm1a proteins (1.0 mg/ml) were added in equal weight at 32°C. Following the instructions, the mixture was then incubated at 32°C for 10 min with constant stirring (500 rpm). To avoid degradation, the formulated Em1a-201VG emulsions were kept at 4°C for less than 2 h until use.

Following the previous description (43), the double emulsion solvent evaporation technique (*w*/*o*/*w*) was utilized for the synthesis of PLGA nanospheres with minor alterations. Briefly, to obtain the organic phase, 50 mg of PLGA (MW: 40,000–75,000 Da, LA/GA: 65/35; Sigma, St. Louis, MO, USA) was firstly dissolved in 1.0 ml dichloromethane (DCM; Sigma) at room temperature. Then, 2.0 ml of 5.0% (*w*/*v*) polyvinyl alcohol (PVA; MW: 31,000–75,000 Da, Sigma) was immediately dropwise added. After vortexing at maximum speed for several minutes until the liquid changed to cloudy, tip sonication was subsequently conducted in a continuous mode (duration time 2 s, interval time 2 s) under the output power of 40 W in an ice bath for about 5 min until the liquid changed to milky white. To synthesize the aqueous phase, 4.0 ml of rEm1a proteins at 1.0-mg/ml concentration were added dropwise. Vortexed again at room temperature for 5 min, the mixture was then tip-sonicated in the same way mentioned above. To obtain the *w*/*o*/*w* emulsions, 2.0 ml of 5.0% (*w*/*v*) PVA was added dropwise, and then tip sonication was again conducted. The obtained *w*/*o*/*w* emulsions were passed through the 0.22-μm filtering membrane (Millipore, Billerica, MA, USA) and centrifuged at 40,000 rpm for 30 min at 4°C. The precipitates were then collected and resuspended in double-distilled water. The solution was then kept at -80°C until it was completely frozen. To fully remove DCM and be preserved, the frozen PLGA nanospheres were completely freeze-dried (Labconco, Kansas City, MO, USA). The Em1a-PLGA nanospheres were then stored in powder form at -20°C and diluted in PBS before use.

As for the CS nanospheres, the ionic gelation technique was conducted as described previously (44). In brief, 100.0 mg of CS (MW: 50–190 kDa; Sigma) was first dissolved in 50.0 ml of 1.0% (*v*/*v*) aqueous solution of acetic acid to obtain 2.0 mg/ml CS solution, and then the pH value was adjusted to 5.0 by 1 mol/L NaOH solution. Ten milliliters of CS solution (2.0 mg/ml) was put into a centrifugal tube, and the tube was sat on a magnetic stirrer (500 rpm) at the temperature of 30°C. Then, 4.0 ml of rEm1a protein (1.0 mg/ml) and 2.0 ml of sodium tripolyphosphate solution (2.0 mg/ml; TPP, Aladdin, Shanghai, China) were then respectively dropwise added. Tip sonication was then carried out in a continuous mode (duration time 4 s, interval time 2 s) under the output power of 50 W in an ice bath for 2 min. The mixture was then centrifuged at 40,000 rpm for 15 min at 4°C to collect the precipitate. After dissolving in double-distilled water and passing through the 0.22-μm filtering membrane, the precipitate was kept at -80°C until the liquids were completely frozen. For long-term preservation, the frozen CS nanospheres were then completely freeze-dried as stated above. The Em1a-CS nanospheres were then stored in powder form at -20°C and diluted by PBS before use.

### Characterization of Nanospheres

To characterize the surface morphology of the synthesized nanospheres, the freeze-dried Em1a-PLGA and Em1a-CS nanospheres were examined through a scanning electron microscope (SEM; SU8010, Hitachi, Tokyo, Japan). To access the average diameter of synthesized nanospheres, five arbitrary nanospheres in SEM images were measured by ImageJ 1.8.0 software (NIH, Bethesda, MD, USA). To evaluate the EE and loading capacity (LC), the supernatant after centrifugation was collected, and the free proteins were evaluated by a BCA method. In addition, the total volume of the collected supernatant was also measured. Then, EE and LC can be calculated according to Formula 1–3.


   (1) Free protein (mg) = Free protein concentration ×Supernatant volume

(2) $$\text{EE }\left(\%\right)=\frac{\text{Total protein}-\text{Free protein}}{\text{Total protein}}\;\times 100\%$$

(3) $$\text{LC }\left(\%\right)=\frac{\text{Weight of nanospheres }-\text{Free protein}}{\text{Weight of nanospheres}}\;\;\times \;100\%$$

The *in vitro* release characteristics of formulated nanospheres were investigated as described previously (45). Freeze-dried nanospheres were first dissolved in PBS (pH 7.4) at 37°C under mild shaking (180 rpm). At an interval of 12 h, nanosphere solutions were centrifuged at 12,000 rpm for 1 min at room temperature, and 20 μl of supernatant was collected and stored at -20°C. The nanospheres that settled at the bottom were resuspended, and the total volume of nanosphere solutions was recorded after each collection time point. After the last collection, samples were determined for protein concentration using the Pierce™ BCA Protein Assay Kit (Thermo Scientific), and absorbance was evaluated by a microplate spectrophotometer (Thermo Scientific). The cumulative release (CR) profile was calculated by Formula 4.


(4) $$\text{CR }\left(\%\right)=\frac{\text{Total volume }\times \text{Protein concentration}}{\text{Total loaded proteins}}\;\times 100\%$$

To determine the toxicity of freeze-dried nanospheres, 35 BALB/c mice were randomly assigned to seven groups (n = 5): Blank (injected with equal volume of PBS), Control (injected with pET-32a vector protein), Em1a (injected with rEm1a protein), Em1a-PLGA (injected with Em1a-PLGA nanospheres), and Em1a-CS (injected with Em1a-CS nanospheres). Through intramuscular injection, each mouse was immunized with a dose of 300 μg of protein (15 mg/kg of body weight), which was three times as usual (5 mg/kg of body weight). Three days later, a booster injection was given using the same strategy. One day later, sera were harvested through retro-orbital blood collection, and the levels of blood urea nitrogen (BUN) and creatinine (Cr) in the harvested sera were investigated using the commercially available kits (Solarbio, Beijing, China). The mental status and physical status of tested mice were observed daily.

### Immunization and Inoculation in Animals

Day-old chicks were randomly divided into 12 groups (40 birds/group) and were vaccinated with multiple intramuscular injections in the leg. For single vaccination, the maximum dosage for each animal was controlled within 500 μl. To evaluate the protective efficacy generated by different preparations, 10 chickens in each experimental group were orally challenged with 5 × 10^4^ *E. mitis* sporulated oocysts (high-dose challenge) at the age of 4 weeks. Simultaneously, 10 chickens in each experimental group were also orally challenged with 3,000 *E. mitis* sporulated oocysts (low-dose challenge) (**Table 1**). Six days after the challenge, all animals were euthanized by intravenously injecting excess phenobarbital under the supervision of the Animal Ethics Committee, Nanjing Agriculture University, China. From the first day to the sixth day after the challenge, feces excreted by each low-dose challenged bird were collected, thoroughly mixed, and stored at 4°C. To evaluate the weight changes, 10 animals (high-dose challenged) from each group were weighed at the age of 2 weeks (before the first immunization), 4 weeks (1 week after the booster immunization), and 5 weeks (1 week after the challenge). The weight gain of each bird was evaluated according to Formula 5.


  (5) $$\text{Coefficient of growth }\left(\%\right)=\frac{\text{Final weight}-\text{Initial weight}}{\text{Initial weight}}\;\times \;100\%$$

**Table 1:** Group and immune procedure in animal experiments.

Group	Bird vaccination ^1^		Bird challenge	
	Treatment (each animal)	Total birds per treatment ^2^	High-dose challenge	Low-dose challenge ^3^
Blank (PBS)	Equal volume of PBS	40	Ten animals were challenged with equal volumes of PBS (0 oocyst)	Ten animals were challenged with equal volumes of PBS (0 oocyst)
Blank (Coccidia)	Equal volume of PBS	40	Ten animals were challenged with 5 × 10^4^ oocysts of purified *E. mitis*	Ten animals were challenged with 3,000 oocysts of purified *E. mitis*
Control	200 μg pET32a vector protein	40		
71VR	Equal volume of Montanide™ ISA-71R VG	40		
201VG	Equal volume of Montanide™ ISA-201VG	40		
PLGA	Equal volume of PLGA nanosphere loading PBS	40		
CS	Equal volume of CS nanosphere loading PBS	40		
Em1a	200 μg Em1a proteins	40		
Em1a-71VR	Em1a-71VG emulsions containing 200 μg Em1a proteins	40		
Em1a-201VG	Em1a-201VG emulsions containing 200 μg Em1a proteins	40		
Em1a-PLGA	Em1a-PLGA nanospheres containing 200 μg Em1a proteins	40		
Em1a-CS	Em1a-CS nanospheres containing 200 μg Em1a proteins	40		

^1^All birds were vaccinated at ages 2 and 3 weeks.

^2^Before first immunization (week 2), five chickens from each group were euthanized to obtain serum samples. One week after the first immunization (week 3), five chickens from each group were sacrificed to obtain the sera and splenic lymphocytes. One week after the booster immunization (week 4), 10 animals were euthanized to separate enough splenic lymphocytes. Ten animals were used for the high-dose challenge, and 10 animals were used for the low-dose challenge.

^3^All birds were challenged at the age of 4 weeks.

PBS, Phosphate-buffered saline; PLGA, poly lactic-co-glycolic acid; CS, chitosan; Em1a, E. mitis 1a protein.

### Antibody and Cytokine Determinations

At the age of 2, 3, and 4 weeks, five vaccinated chickens were anesthetized, and blood was harvested by heart puncture. The sera were subsequently separated and stored at -20°C. Based on the previous report (46), enzyme-linked immunosorbent assays (ELISAs) were conducted to investigate Em1a-specific serum antibody levels. Briefly, each well of the 96-well plates (Costar, Cambridge, MA, USA) was coated with 1 μg recombinant Em1a proteins (diluted to 10 μg/ml with carbonate-bicarbonate buffer pH 9.6) overnight at 4°C. After being washed thrice in TBST, nonspecific binding sites were blocked with TBST containing 5% (*w*/*v*) skimmed milk powder at 37°C for 2 h. Rinsed in TBST for 5 min, the wells were then incubated with sera (1:100 dilutions) from vaccinated chickens at 37°C for 1 h. Rinsed again in TBST, each well was incubated with horseradish peroxidase (HRP)-conjugated anti-chicken IgY (1:8,000 dilutions; Abcam, Cambridge, UK) at 37°C for 1 h. After rinsing in TBST for 5 min, each well was added with 100 μl 3,3’,5,5’-tetramethylbenzidine (TMB; Tiangen, Beijing, China) to develop colors at room temperature. After a 15-min incubation, 100 μl of newly prepared H_2_SO_4_ (2 M) was added to stop the reactions. The absorbance was detected at OD450 by a microplate photometer (Thermo Scientific). Each group consisted of five replications, and each replication was measured once.

To investigate cytokine levels in the sera collected from 4-week-old birds, commercially available ELISA kits (Enzyme-linked Biotechnology, Shanghai, China) were used. Strictly following the manufacturer’s instructions, the concentrations of interferon-gamma (IFN-γ), interleukin 4 (IL-4), transforming growth factor β (TGF-β), IL-6, IL-10, and IL-17 in animals’ sera were investigated. Cytokine levels were calculated by referring to the standard curves constructed from known concentrations of recombinant chicken cytokines. Each group consisted of five replications, and each replication was measured once.

### Proliferation of Splenic Lymphocytes

Seven days after the booster immunization (before challenge), five birds from each group were euthanized, and the lymphocytes were isolated by using the separation solution (TBD, Tianjin, China) according to a previous report (47). To analyze the proliferation of splenic lymphocytes, 10^5^ cells were diluted in 100 μl of Gibco™ Dulbecco’s modified Eagle’s medium (DMEM; Invitrogen Biotech) containing 20% (*v*/*v*) fetal bovine serum (FBS) and 20 μg/ml of rEm1a protein and were added to the well of 96-well plates. Cultured at 37°C in a 5% (*v*/*v*) CO_2_ atmosphere for 72 h, splenic lymphocytes in each well were then incubated with 10 μl of Cell Counting Kit 8 (CCK-8) reagent (Beyotime Biotech, Shanghai, China) based on the manufacturer’s instructions. After a 4-h incubation under the same conditions, the absorbance of each well was evaluated at OD450 using a microplate photometer. Each group consisted of five replications, and each replication was conducted three times.

### Detection of the Proportions of CD4^+^ and CD8^+^ T Lymphocytes

To obtain splenic lymphocytes, five chickens from each group were respectively euthanized 1 week after the first immunization (week 3) and 1 week after booster immunization (week 4). The lymphocytes were then separated as described in 2.9. To analyze the percentages of CD4^+^ T lymphocyte subsets, obtained splenic lymphocytes (10^6^ cells suspended in 100 μl PBS) were dually stained with anti-chicken CD3-FITC (Southern Biotech, Birmingham, AL, USA) and CD4-APC (Southern Biotech) for 30 min at 4°C in the dark. As for the proportions of CD8^+^ T-lymphocyte subsets, 10^6^ splenic lymphocytes suspended in 100 μl PBS were dually stained with anti-chicken CD3-FITC and CD8-PE (Southern Biotech). After being washed thrice in PBS, lymphocytes were characterized by flow cytometry (Beckman Coulter Inc., Brea, CA, USA). Before cell sorting, fluorescence compensation was conducted using the fluorescence minus one (FMO) control. Each group consisted of five replications, and each replication was measured once.

### Parasite Burden in Chickens

To determine *E. mitis* burdens in animals, 200 mg of feces were lysed for the genomic DNA, according to the guidelines (Omega Bio-Tek). The extracts were kept at -20°C until use. Based on the single-copy sequence originating from sequence-characterized amplified region markers, absolute quantitative real-time PCR (qPCR) was performed according to the published research (48). According to the instructions of the High-Fidelity Master Mix, the plasmids containing the amplified fragments were also constructed by the primers: 5′-GCAGGGCAGGCAGGGTAG-3′ and 5′-GCACGGCAGGCTCAGAAA-3′ (GeneBank: AY571506.1). The PCR reaction was carried out in a volume of 25 μl under the following conditions: 95°C for 5 min, followed by 35 cycles of 95°C for 30 s, 60.6°C for 30 s, and 72°C for 80 s; and a final extension at 72°C for 5 min was also conducted. The PCR amplicons were then subcloned to a linearized pMD-19T vector (Takara Biotechnology). For qPCR amplifications, 10 μl of PerfectStart^®^ Green qPCR SuperMix (TransGen Biotech, Beijing, China), 0.4 μl of each primer, 0.4 μl of 50× ROX Reference Dye 2, 1.0 μl of genomic DNA extracts, and double-distilled water were mixed to a 20.0-μl volume. Amplifications were then carried out using an Applied Biosystem 7500 (Life Technologies, Carlsbad, CA, USA) by the following program: 95°C for 30 s, followed by 40 cycles of 95°C for 10 s and 60°C for 10 s. The melt-curve analysis was also conducted at the end of the reactions. Before further analysis, one uniform peak of the melting curve was confirmed in each amplification. Each group consisted of 10 replications, and each replication was measured once.

### Statistical Analysis

Statistical analysis was analyzed by GraphPad Prism 8.0 (GraphPad Software, San Diego, CA, USA). One-way analysis of variance (ANOVA) was performed for antibody analysis, cytokine analysis, flow cytometry analysis, proliferation analysis, weight analysis, and parasite burden analysis. Comparisons among Em1a, Em1a-71VR, Em1a-201VG, Em1a-PLGA, and Em1a-CS groups were conducted by ANOVA alongside Bonferroni correction. Values were shown as mean ± standard deviation (SD), and a significant difference was considered at *p* < 0.05. In addition, flow cytometric analysis was estimated by CytExpert 2.3 software (Beckman Coulter Inc., Brea, CA, USA).

## Results

### Cloning, Expression, Purification, and Immunoblot Analysis of rEm1a Proteins

**Figure 1:**
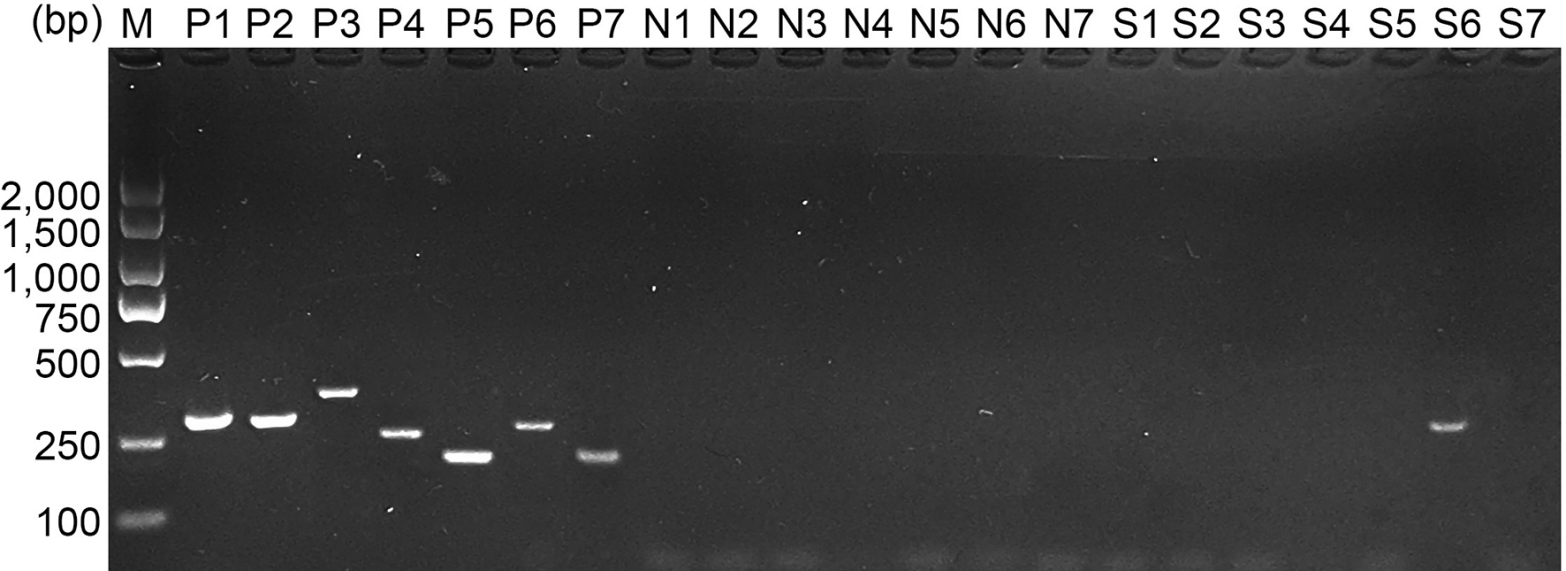
Molecular identification of *E. mitis* sporulated oocysts. *Eimeria* species were detected by PCR amplification based on the internal transcribed spacer 1 (ITS1) sequence. Line M: DL2,000 marker; Lines P1–P7: positive control for *E. acervulina*, *E. brunetti*, *E. necatrix*, *E. tenella*, *E. maxima*, *E. mitis*, and *E. praecox*; Lines N1–N7: negative control for *E. acervulina*, *E. brunetti*, *E. necatrix*, *E. tenella*, *E. maxima*, *E. mitis*, and *E. praecox*; Lines S1–S7: DNA extracts for *E. acervulina*, *E. brunetti*, *E. necatrix*, *E. tenella*, *E. maxima*, *E. mitis*, and *E. praecox* detection.

**Figure 2:**
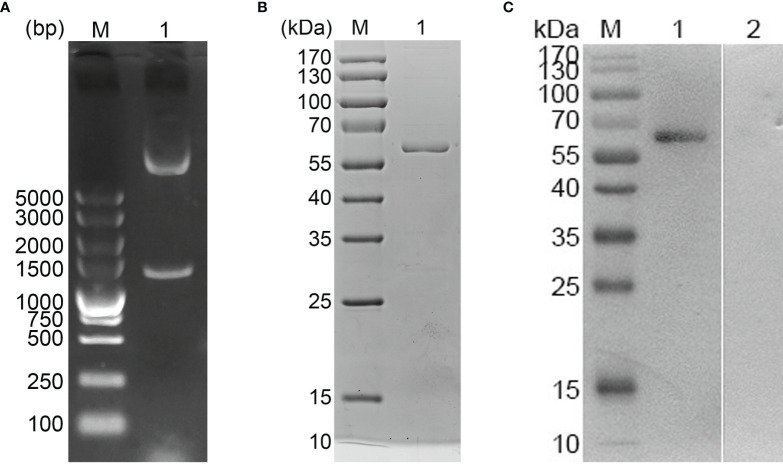
**(A)** Double digestion analysis. Line M: DL5,000 marker; Line 1: Double digestion of the constructed pET-32a-Em1a plasmid by *Kpn*I and *Eco*RV. **(B)** SDS-PAGE analysis. Line M: Molecular weight (MW) marker proteins. Line 1: purified Em1a proteins. **(C)** Western blot analysis of purified Em1a proteins. Line M: MW marker proteins. Line 1: Purified Em1a proteins were detected by sera from *E mitis*-infected chickens; Line 2: Purified Em1a proteins were detected by sera from control chickens.

*E. mitis* oocysts were proven to be pure according to the PCR assay (**Figure 1**), and recombinant pET-32a-Em1a plasmid was successfully constructed. The double enzyme digestion was also conducted with *Kpn*I and *Eco*RV to verify the newly constructed plasmid, yielding two fragments, 1,256 bp and 5,868 bp in theory (**Figure 2A**, Lane 1). In addition, DNA sequencing also indicated that the pET-32a-Em1a plasmid was correctly constructed. According to pET-32a sequence landmarks, the rEm1a protein expressed by the pET-32a-Em1a plasmid-transformed cells contains the specific His-tag (16.49 kDa). Theoretically, the molecular weight of the rEm1a protein was 61.10 kDa (**Figure 2B**, Lane 1). The endotoxin level fell to 0.1 EU/ml after removing the endotoxin from purified rEm1a proteins. Based on the Western blot assay (**Figure 2C**, Lane 1), rEm1a proteins could be detected by anti-*E. mitis* serum generated by host humoral immune responses, indicating a satisfactory antigenicity of the rEm1a protein.

### Characteristics of the Formulated Nanospheres

**Figure 3:**
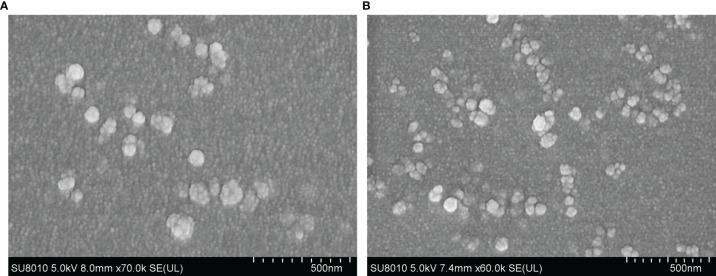
Characterization of Em1a-PLGA **(A)** and Em1a-CS **(B)** nanospheres. Evaluated using SEM, Em1a-PLGA nanospheres were prepared with double emulsion solvent evaporation technique, while the Em1a-CS nanospheres were synthesized by the ionic technique. Bar represented 500 nm.

SEM images showed uniform spherical Em1a-PLGA nanospheres with a rough surface. The average size was 111.54 ± 22.09 nm (n = 5) in diameter (**Figure 3A**). As illustrated in **Figure 3B**, Em1a-CS nanospheres were spherical with small particles on their surface, and the mean diameter was about 82.43 ± 11.35 nm (n = 5). Furthermore, the EE and LC of the Em1a-PLGA and Em1a-CS nanospheres were also evaluated. Prepared by 5.0% PVA, the EE of Em1a-PLGA nanospheres reached 68.23% (n = 3) when the concentration of rEm1a proteins reached 1.0 mg/ml. Synthesized by 2.0 mg/ml of TPP and 1.0 mg/ml of purified rEm1a, the EE of Em1a-CS nanospheres reached 46.76% (n = 3). Based on three independent trials, the LC of Em1a-PLGA and Em1a-CS nanospheres was 0.92% and 4.90%, respectively.

**Figure 4:**
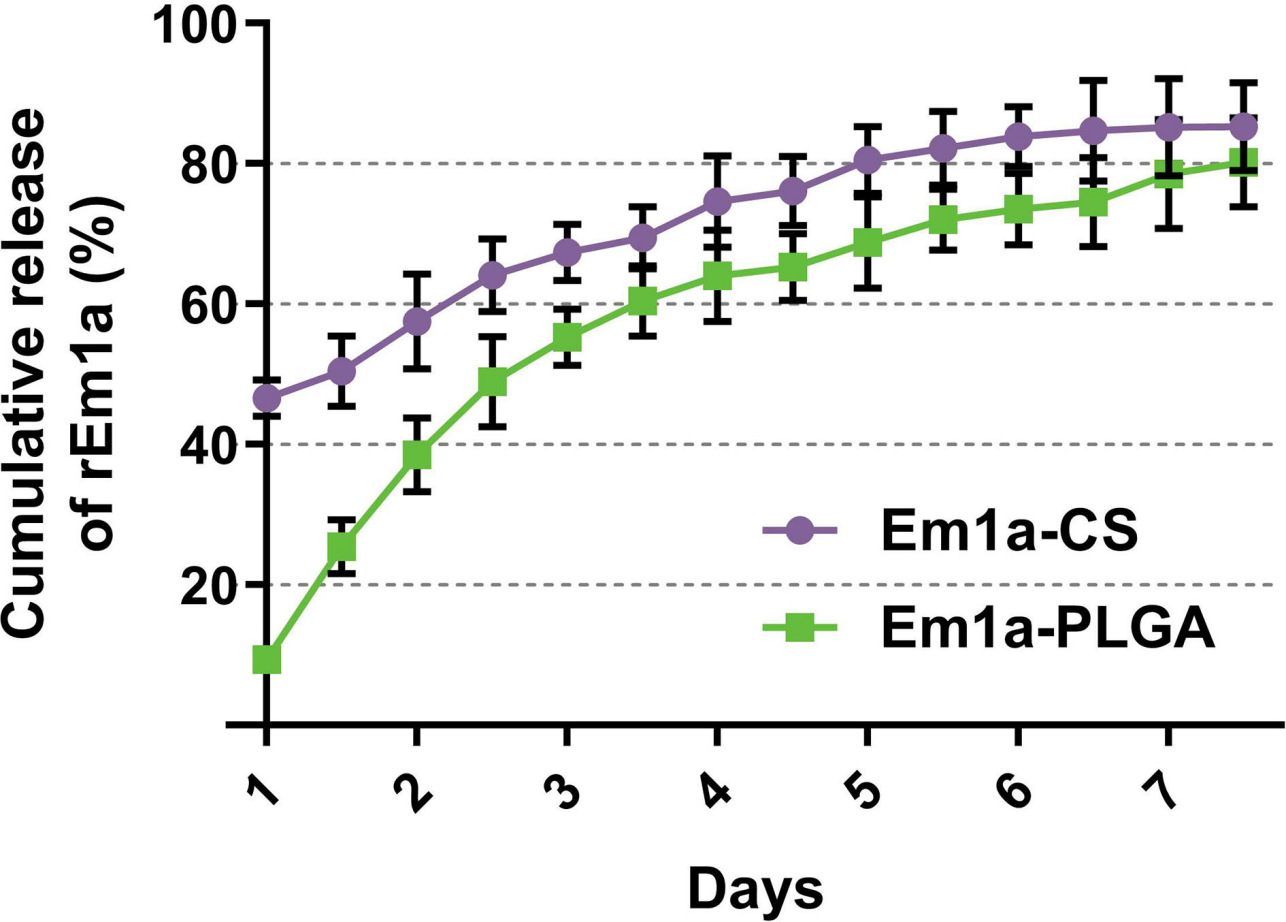
*In vitro* release of recombinant Em1a proteins from Em1a-PLGA and Em1a-CS nanospheres. The concentrations of free proteins were investigated by the BCA assay. Three independent experiments were carried out, and each sample was measured once. Values were presented as the mean ± SD (n = 3).

The release profile of the rEm1a proteins from PLGA and CS nanospheres was measured, showing a sustained slow release over a 7-day period. As demonstrated in **Figure 4**, the fast dissolution properties of Em1a-CS nanospheres were evaluated, and the release of rEm1a protein from Em1a-CS nanospheres was 46.59% of the total encapsulated antigens at 0 h. However, the Em1a-CS nanospheres generated a steadier release when compared with the Em1a-PLGA nanospheres within the first 2 days. After the fourth day, the release curve of Em1a-PLGA nanospheres became flat, while the release profile of Em1a-CS nanospheres turned to be smooth after the third day.

**Figure 5:**
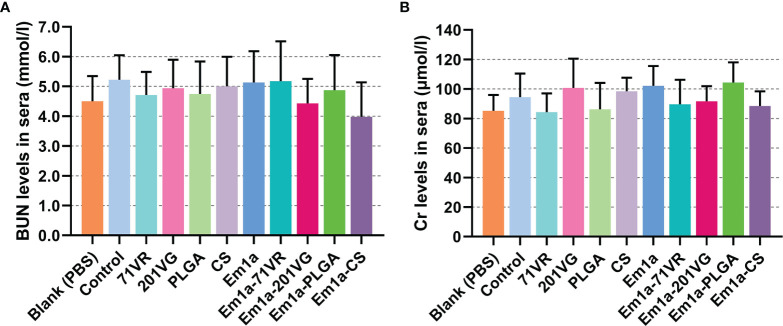
The toxicity of recombinant Em1a proteins and its nanospheres. Based on the urease-indophenol and sarcosine oxidase method, the levels of BUN **(A)** and Cr **(B)** were investigated based on the commercial kits. Each sample was tested once, and values were estimated using one-way ANOVA followed by Dunnett’s test. Comparisons among Em1a, Em1a-71VR, Em1a-201VG, Em1a-PLGA, and Em1a-CS group were conducted by ANOVA following Bonferroni’s correction. Values were presented as the mean ± SD (n = 5).

In veterinary applications, toxicity is one of the major concerns when using nanospheres and even nontoxic ones with biodegradable characteristics. Therefore, we tested the toxicity of Em1a-PLGA and Em1a-CS nanospheres in mice (**Figure 5**). The levels of BUN and Cr in mice were maintained in an acceptable range with no significance (*p* > 0.05), indicating that rEm1a protein and its nanospheres did not affect the health status of animals. As for mental status and physical status, all mice were in mental health, and no adverse reaction occurred after the vaccination. All of these observations demonstrated that the rEm1a protein and its synthesized nanospheres were nontoxic to animals.

### Measurements of Antibody and Cytokine Levels

**Figure 6:**
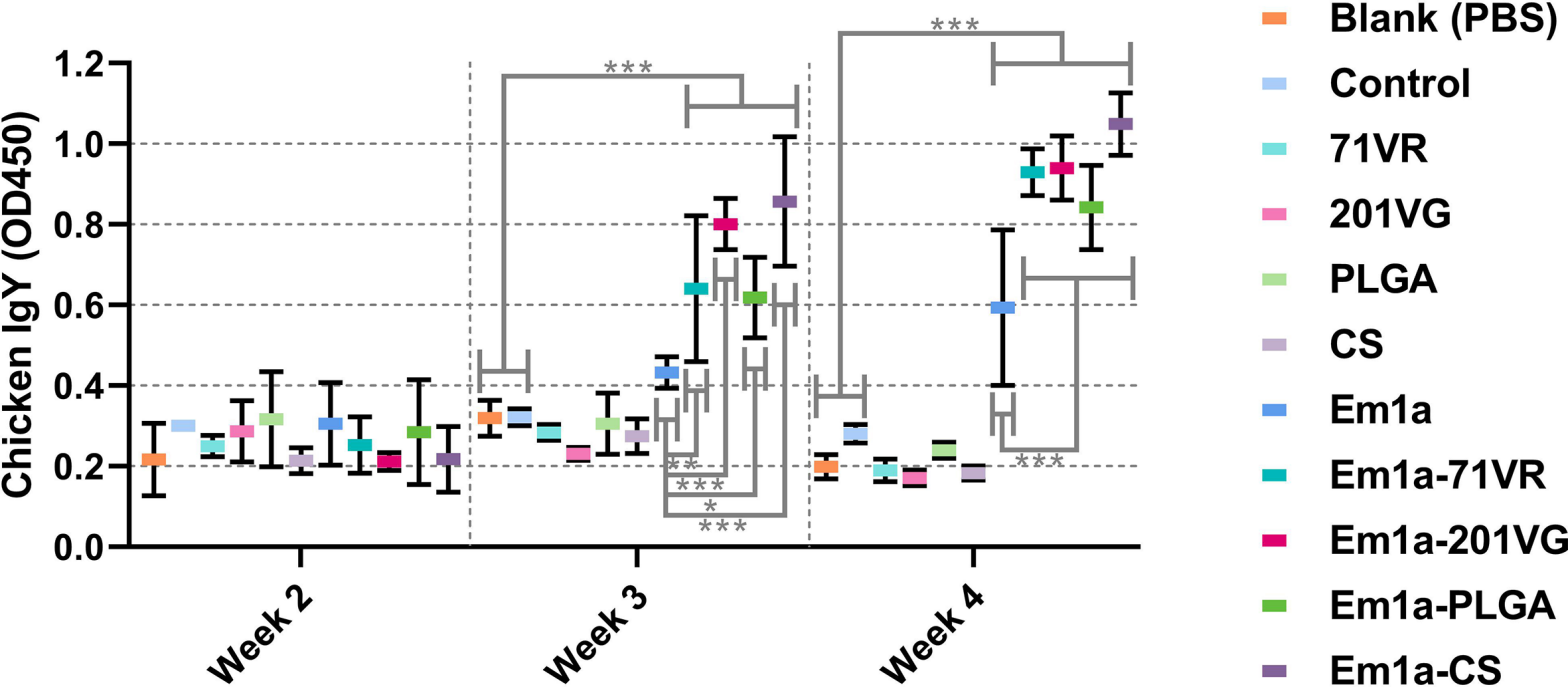
Determination of Em1a-specific antibody in the animals’ sera. Each sample was tested once, and values were estimated using one-way ANOVA followed by Dunnett’s test. Comparisons among Em1a, Em1a-71VR, Em1a-201VG, Em1a-PLGA, and Em1a-CS group were conducted by ANOVA following Bonferroni’s correction. Values were presented as the mean ± SD (n = 5). In all studies, *p < 0.05, **p < 0.01, and ***p < 0.001.

To evaluate antibody-mediated immunity, birds were intramuscularly injected with 200 μg of purified rEm1a antigen or corresponding nanospheres. Serum samples were harvested at the age of 2 weeks (before the first immunization), 3 weeks (1 week after the first immunization), and 4 weeks (1 week after the second immunization). We investigated the ability of rEm1a to induce humoral responses in birds by quantifying rEm1a-specific antibodies using ELISAs. As demonstrated in **Figure 6**, immunizations with Em1a-71VR, Em1a-201VG, Em1a-PLGA, and Em1a-CS elicited significantly higher IgY levels than that of rEm1a-immunized chickens at the age of 3 and 4 weeks. When compared with the blank or control group 1 week after the first or booster immunization, no rEm1a-specific antibody was detected in serum samples obtained from the 71VR, 201VG, PLGA, and CS groups (*p* > 0.05) (**Figure 6**).

**Figure 7:**
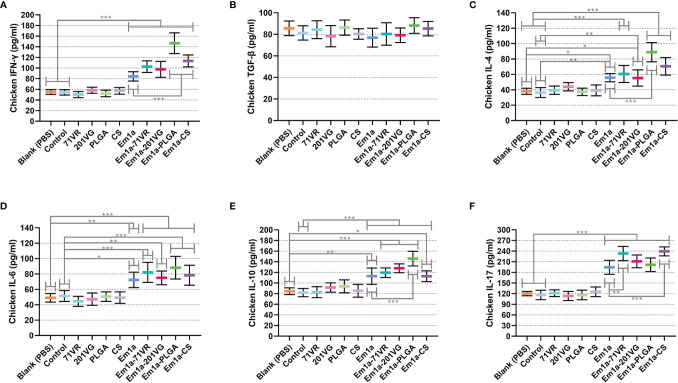
Determination of cytokine secretions in the animals’ sera. The concentrations of IFN-γ **(A)**, TGF-β **(B)**, IL-4 **(C)**, IL-6 **(D)**, IL-10 **(E)**, and IL-17 **(F)** were investigated by ELISA kits. Each sample was tested once, and values were estimated using one-way ANOVA followed by Dunnett’s test. Comparisons among Em1a, Em1a-71VR, Em1a-201VG, Em1a-PLGA, and Em1a-CS group were conducted by ANOVA following Bonferroni’s correction. Values were presented as the mean ± SD (n = 5). In all studies, *p < 0.05, **p < 0.01, and ***p < 0.001.

Sera were isolated from five chickens in each group 7 days after the booster immunization. Cytokine secretions were then detected by commercially available ELISA kits. As illustrated in **Figure 7A**, evidently higher levels of IFN-γ could be detected in Em1a, Em1a-71VR, Em1a-201VG, Em1a-PLGA, and Em1a-CS groups when compared to the blank or control group. In addition, Em1a-PLGA- or Em1a-CS-immunized mice generated higher levels of IFN-γ than those in the rEm1a-immunized group (*p* < 0.001) (**Figure 7A**). As for TGF-β secretion, no statistical difference was observed between the rEm1a-loaded vaccines and rEm1a-immunized group (*p* > 0.05) (**Figure 7B**). The Em1a-PLGA-immunized group produced higher levels of IL-4 than the rEm1a-immunized group (*p* < 0.001) (**Figure 7C**), whereas no difference in IL-6 secretion was observed between the Em1a-PLGA- and rEm1a-immunized groups (**Figure 7D**). However, enhanced secretions of IL-4 and IL-6 could be detected in birds immunized with rEm1a-loaded vaccines compared to the blank or control group (**Figures 7C, D** **)**. As for IL-10, IL-10 secretions in the Em1a-PLGA group were remarkably increased (*p* < 0.001) when compared to only rEm1a-immunized birds (**Figure 7E**). Furthermore, Em1a-71VR emulsions (*p* < 0.01) and Em1a-CS nanospheres (*p* < 0.001) could elicit higher productions of IL-17 than the rEm1a proteins (**Figure 7F**). Notably, compared to the blank or control group, birds immunized with rEm1a-loaded preparations could produce significantly higher levels of IL-10 and IL-17 when compared to the blank or control group (**Figures 7E, F** **)**.

### Determination of Lymphocyte Proliferation

**Figure 8:**
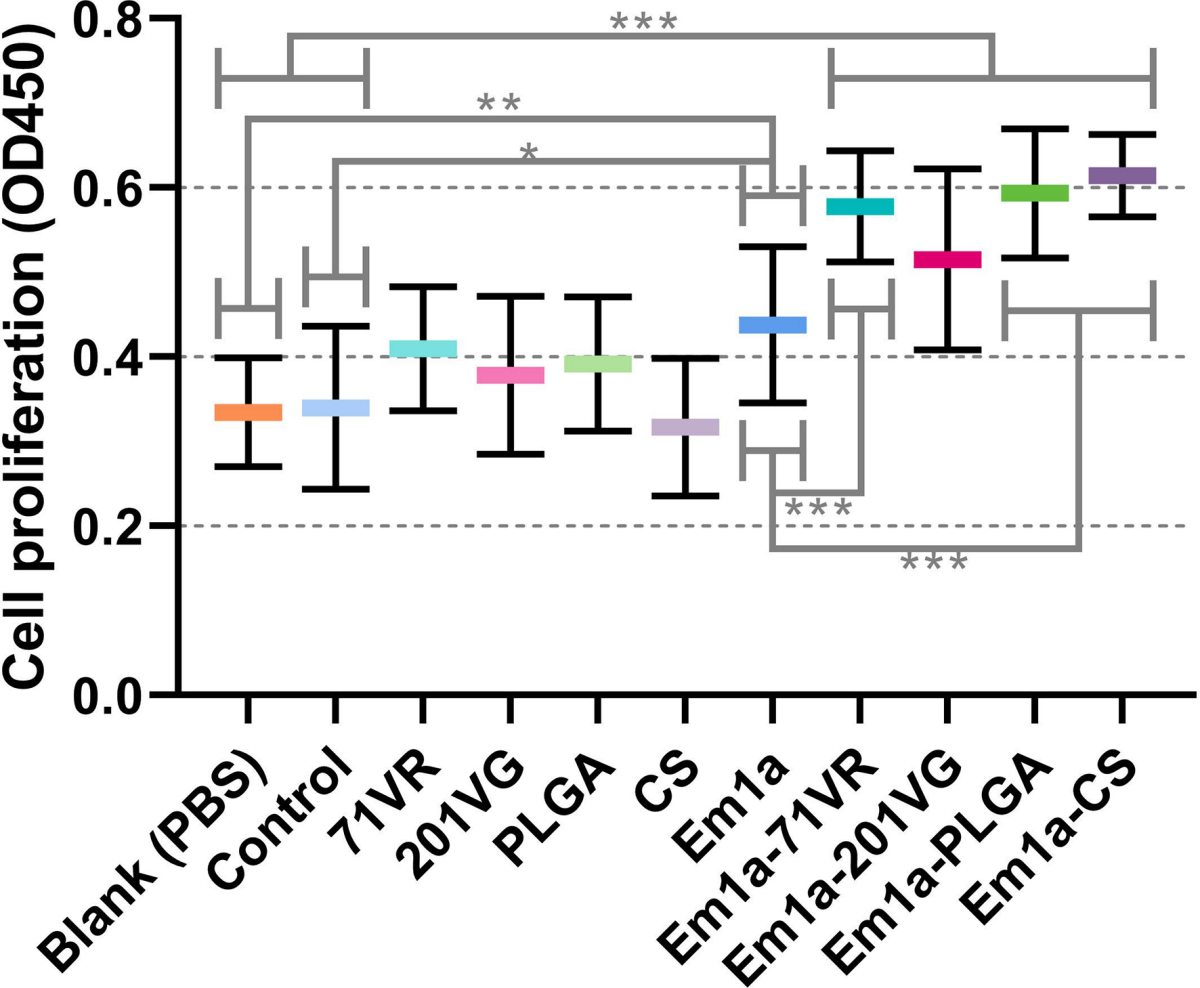
The proliferation of splenic lymphocytes isolated from chickens. Five animals in each group were sacrificed, and the splenocytes from each animal were independently tested three times (n = 5). Values were estimated using one-way ANOVA followed by Dunnett’s test. Comparisons among Em1a, Em1a-71VR, Em1a-201VG, Em1a-PLGA, and Em1a-CS group were conducted by ANOVA following Bonferroni’s correction. Values were presented as the mean ± SD (n = 15). In all studies, *p < 0.05, **p < 0.01, and ***p < 0.001.

Mature dendritic cells play an essential role in the activation and proliferation of T lymphocytes. To investigate the effects of rEm1a protein and its encapsulations on splenic lymphocyte proliferation, splenocytes were harvested and evaluated using the CCK-8 reagent. As demonstrated in **Figure 8**, immunizations with Em1a-71VR emulsions, Em1a-PLGA nanospheres, and Em1a-CS nanospheres promoted splenocyte proliferation when compared with unencapsulated rEm1a antigen (*p* < 0.001). Furthermore, significant differences in splenocyte proliferation were observed in any form of rEm1a-loaded preparations when compared to the blank or control group (**Figure 8**).

### Cellular Immune Responses in Splenic Lymphocytes

**Figure 9:**
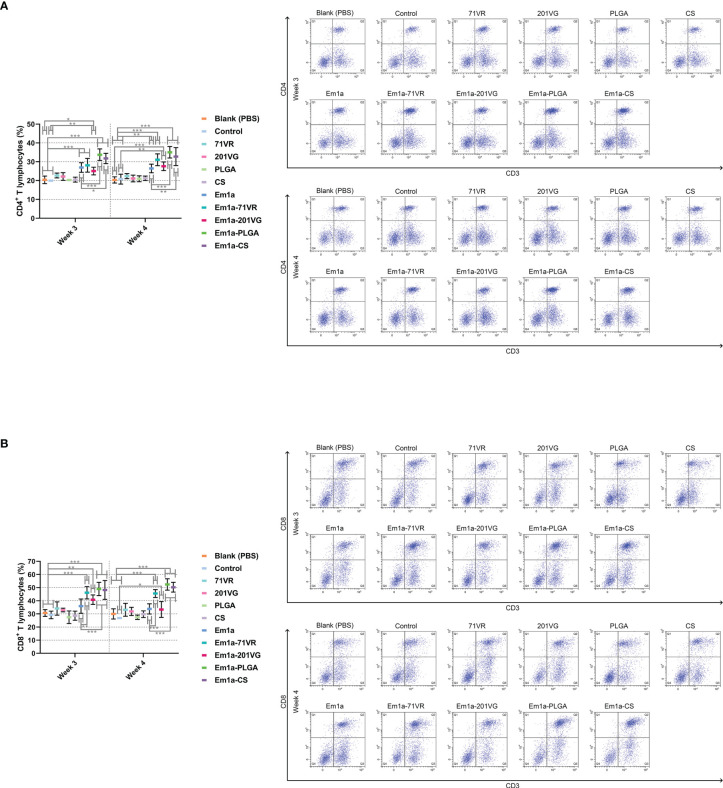
Flow cytometry analysis of CD4^+^ **(A)** and CD8^+^ **(B)** T lymphocytes in splenocytes at the age 3 and 4 weeks. Chickens from each group were sacrificed, and the splenic lymphocytes were harvested. Based on the FMO control, adequate compensation was conducted before cell sorting. Each sample was measured once, and values were estimated using one-way ANOVA followed by Dunnett’s test. Comparisons among Em1a, Em1a-71VR, Em1a-201VG, Em1a-PLGA, and Em1a-CS group were conducted by ANOVA following Bonferroni’s correction. Values were presented as the mean ± SD (n = 5). In all studies, *p < 0.05, **p < 0.01, and ***p < 0.001.

Seven days after the first and second immunization, lymphocytes were harvested from the spleens. Flow cytometry analysis showed that immunization with Em1a-PLGA and Em1a-CS nanospheres induced higher levels of CD4^+^ T cells after the first and booster immunizations than that with rEm1a protein alone (**Figure 9A**). All birds immunized with rEm1a-loaded preparations could generate significantly higher levels of CD4^+^ T cells after the booster immunization (at the age of 4 weeks) when compared to the blank or control group (**Figure 9A**). Compared to the unencapsulated rEm1a protein, vaccination with Em1a-71VR emulsions, Em1a-PLGA nanospheres, and Em1a-CS nanospheres notably increased the expression of CD8 molecules on CD3^+^ T lymphocytes at the age of 3 and 4 weeks (**Figure 9B**). In addition, no significant difference in CD8 expressions on splenocytes was observed across any rEm1a-unencapsulated groups (*p* > 0.05) (**Figure 9B**).

### Growth Performance and Protective Efficacy

**Figure 10:**
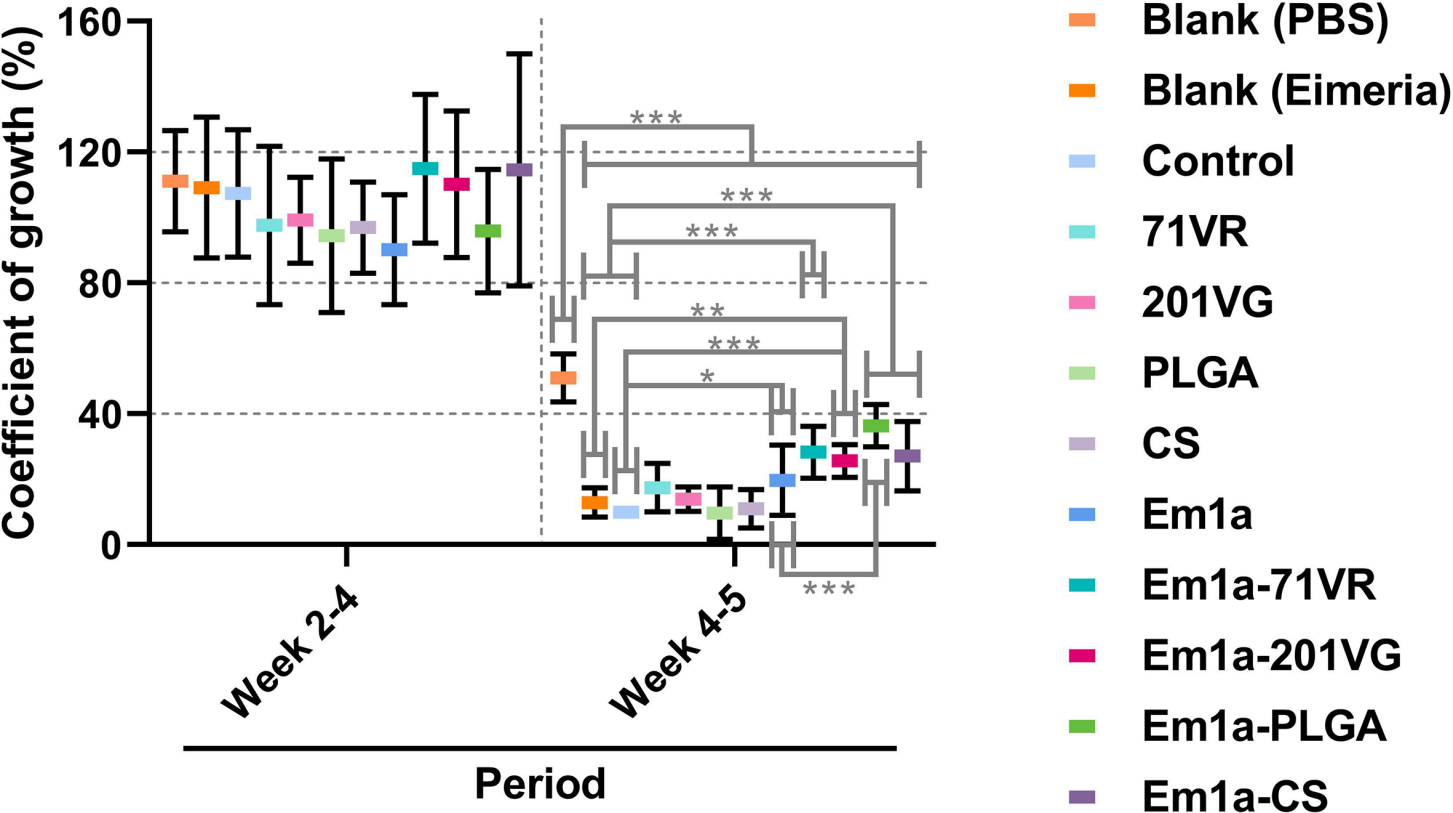
The coefficient of growth in vaccinated chickens. Each animal was orally challenged with 5 × 10^4^ purified *E. mitis* oocysts 1 week after the booster immunization. Animals were weighed at the age of 2 weeks (before the first immunization), 4 weeks (1 week after the booster immunization), and 5 weeks (1 week after the challenge), and the coefficient of growth was calculated. Each animal was measured once, and values were estimated using one-way ANOVA followed by Dunnett’s test. Comparisons among Em1a, Em1a-71VR, Em1a-201VG, Em1a-PLGA, and Em1a-CS group were conducted by ANOVA following Bonferroni’s correction. Values were presented as the mean ± SD (n = 10). In all studies, *p < 0.05, **p < 0.01, and ***p < 0.001.

Ten chickens orally challenged with 5 × 10^4^ *E. mitis* oocysts were selected for recording growth performance. All challenged birds survived throughout the whole trial. Birds were weighed at different time points, and the growth efficiency was calculated. As shown in **Figure 10**, all immunized chickens exhibited similar (*p* > 0.05) body weight gain during the vaccination period (weeks 2–4). However, all challenged chickens exhibited an inhibited growth pattern compared to unchallenged birds (*p* < 0.001). Among all challenged groups, the Em1a-PLGA group (*p* < 0.001), but not other groups immunized with rEm1a-loaded preparations (*p* > 0.05), elicited significantly higher growth efficiency than the Em1a group (**Figure 10**). All of these findings highlight the importance of Em1a-PLGA nanospheres in growth performance against *E. mitis* infections.

**Figure 11:**
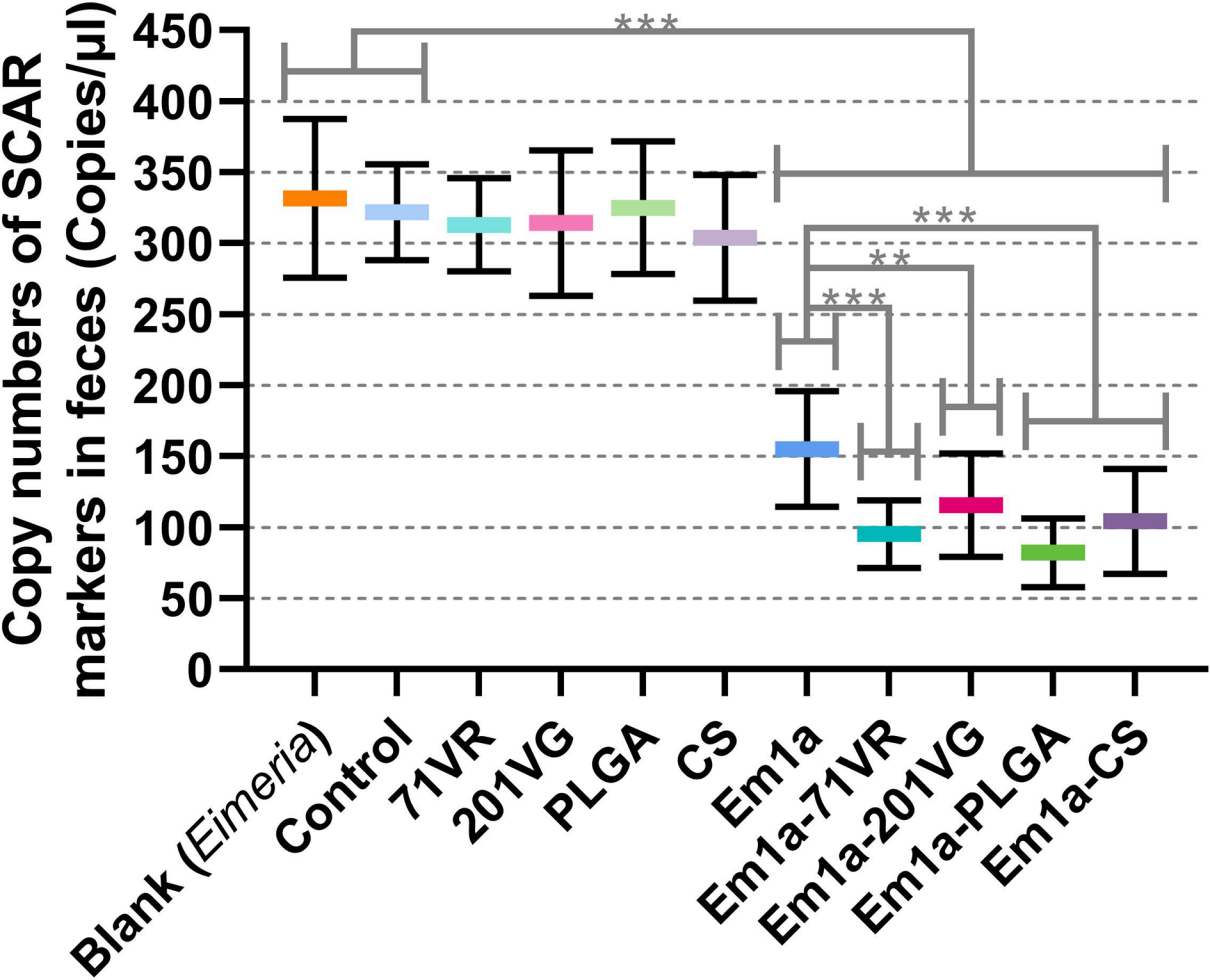
*E. mitis* oocyst burdens in the feces of immunized chickens. Each animal was orally challenged with 3,000 purified *E. mitis* oocysts 1 week after the booster immunization. Six days later, feces excreted by each animal were collected for DNA extractions, and each extract was run in triplicate (n = 10). Values were estimated using one-way ANOVA followed by Dunnett’s test. Comparisons among Em1a, Em1a-71VR, Em1a-201VG, Em1a-PLGA, and Em1a-CS group were conducted by ANOVA following Bonferroni’s correction. Values were presented as the mean ± SD (n = 30). In all studies, **p < 0.01 and ***p < 0.001.

To evaluate the immune protection provided by rEm1a preparations, birds were grouped and orally challenged with 3,000 *E. mitis* oocysts. All immunized chickens survived after *E. mitis* infection, and oocyst output in feces was analyzed 6 days after challenge infection. As shown in **Figure 11**, compared with the Em1a group, *E. mitis* oocyst output was significantly reduced in the Em1a-71VR, Em1a-201VG, Em1a-PLGA, and Em1a-CS groups, indicating that the encapsulations enhanced the immunoprotection of rEm1a proteins. In addition, the Em1a-PLGA group has the lowest oocyst output in feces after the challenge, suggesting the strongest anti-*E. mitis* effect of Em1a-PLGA nanospheres among the tested preparations (**Figure 11**). All of these observations suggested the efficiency of encapsulations of the rEm1a antigen in enhancing the protective immunity against *E. mitis*.

## Discussion

Avian coccidiosis induced by single or mixed infections of *Eimeria* species can cause substantial economic losses to the poultry industry (12, 49). Vaccine immunization to induce robust protective immune responses is an excellent alternative strategy against coccidiosis in poultry. Currently, there has been a growing interest in developing vaccines that use minimal components from pathogens. Notably, vaccines designed based on the recombinant versions of *Eimeria* antigens could carry the immunogenicity of pathogens (50). In the present study, the prokaryotic expression system was constructed to express rEm1a proteins, and the resulting antigen exerted satisfactory immunogenicity by Western blot analysis. The rEm1a antigen was then entrapped in nanomaterials and emulsions. The results indicated that Em1a-PLGA and Em1a-CS nanospheres with a satisfactory release profile were spherical and nontoxic. Furthermore, by evoking humoral and cellular immunity, immunization with corresponding nanospheres recovered growth performance and inhibited oocyst output in *E. mitis*-challenged birds. These results suggested that nanospheres loaded with rEm1a proteins could be an efficient approach to combating *E. mitis* infections.

Nanotechnology-based vaccines offer the possibility to act as efficient and safe alternatives to traditional peptide-based subunit vaccines (51, 52), and numerous techniques nowadays have been applied to formulate nanovaccines (46, 53). Here, we encapsulated the rEm1a antigen in PLGA and CS nanospheres using double emulsion solvent evaporation and ionic gelation technique, respectively. Synthesized Em1a-PLGA and Em1a-CS nanospheres exhibited acceptable levels of EE and LC. The mean diameters of the formulated nanospheres were around 100 nm. Recent findings have revealed that the ability of nanospheres to penetrate cells depends on their size (54), and nanospheres with a diameter of around 100 nm are easier to be absorbed by HeLa cells than those sized around 1,000 nm in diameter (55). It was noteworthy that the EE and LC of formulated nanospheres varied in previous studies. The EE of PLGA-rEm14-3-3 nanospheres reached 89.35% ± 1.18%, while the EE of CS-rEm14-3-3 nanospheres was 83.46% ± 1.57% (56). With similar procedures, the EE of PLGA-rEtTA4 nanospheres reached 82.40% ± 0.06%, while the LC of PLGA-rEtTA4 nanospheres was 2.00% ± 0.01% (46). However, the EE of synthesized CS-PLGA-rOmp22 nanospheres in another paper was approximately 55%, while the LC was approximately 0.94% (57). Such differences may be related to different encapsulation methods or specific encapsulated antigens (44, 53). Further investigations are needed to illustrate the impacts of encapsulated proteins and procedures on EE and LC of synthesized nanospheres.

The sustained release of antigens was an attractive property for the vaccines, as this could decrease immunization times and increase the antigen presentation of antigens to antigen-presenting cells (APCs). As suggested by SEM images, both Em1a-PLGA and Em1a-CS nanospheres were spherical in appearance. They seemed to be composed of many small elements, and such structures may play an essential role in preventing significant degradation. In the present study, we observed a slow-release profile of Em1a-CS nanospheres compared with Em1a-PLGA nanospheres. As a cationic polymer, CS can bind to the surface of cells, resulting in prolonged residence (58). However, the initial burst release was observed in Em1a-CS nanospheres on the first day, and such property may be driven by size, molecular weight, polarity, and even encapsulated antigens of nanospheres (59). Notably, the wide applications of nanospheres in drugs and vaccines are often limited for their toxicity (60). DCM was considered poisonous and difficult to remove by evaporation (61). Given this, we fully freeze-dried the Em1a-PLGA and Em1a-CS nanospheres to remove DCM and eradicate the toxicity. As expected, no adverse reaction occurred in immunized mice, and each animal maintained good mental status, suggesting that the prepared nanospheres were nontoxic to animals.

The critical roles of antibody responses in resisting chicken coccidiosis have been confirmed (62). By preventing pathogens from attaching to host cells, antibodies against pathogen antigens showed great ability in resisting the replications of *Eimeria* species (63). In the current study, high titers of Em1a-specific IgY antibodies were detected in birds immunized with rEm1a-loaded preparations after boosting twice. Our results gave credit to the idea that the rEm1a protein possessed good immunogenicity, and the rEm1a-loaded preparations could induce systemic antibody responses.

Cytokines are crucial in the differentiation of naive T cells into effector cells (64) and play an important role in host immunity against coccidiosis (3, 7). Mainly secreted by CD4^+^ helper T (Th)1 cells, IFN-γ plays a vital role in activating Th1 cells and inhibiting the replications of *Eimeria* species (65, 66). In this study, increased Th1 cytokine productions were observed in birds immunized with the rEm1a-loaded preparations after the booster vaccination, emphasizing that the Th1-type immunity was generated. Furthermore, Th2 immunity is also indispensable for host immunity to combat *Eimeria* parasites (67). IL-4 is predominantly generated by CD4^+^ Th2 cells and CD4^+^ follicular helper T (Tfh) cells (68). Enhanced IL-4 levels were observed in the current research, indicating that rEm1a-loaded vaccines might activate Th2-related and Tfh-related immunity. Moreover, CD4^+^ Th2 cells differentiate into CD4^+^ Th9 cells with the presence of TGF-β (69, 70), and CD4^+^ Th9 cells can also be induced by a large amount of IL-4 (69, 70). In response to parasitic infections (71), activated CD4^+^ Th9 cells can generate the anti-inflammatory cytokine IL-10, which is involved in the maintenance and reestablishment of host immunity (72). However, mainly secreted by inducible regulatory T (iTreg) cells, TGF-β is engaged mostly in immune suppression (73). In the current study, immunized birds exert enhanced secretion patterns of IL-10 and TGF-β, suggesting that Th9-related immune responses were essential in host immunity against coccidiosis. IL-17 is predominantly produced by CD4^+^ Th17 cells, and three IL-17s (IL-17A, IL-17D, and IL-17F) are found to engage in anti-*Eimeria* infections (74, 75). A previous report revealed that the mRNA expression level of IL-17A was significantly upregulated in intestinal intraepithelial lymphocytes after *E. acervulina* or *E. maxima* infections, indicating the association of IL-17A with *Eimeria* infections (76, 77). Enhanced IL-17 secretion was identified in rEm1a preparation-immunized birds, demonstrating a potential role of Em1a in regulating Th17 immune responses against *E. mitis*. In addition, IL-6 can promote the specific differentiation of naive T cells, thereby linking innate and adaptive immunity (78). IL-6 in combination with TGF-β is critical for CD4^+^ Th17 differentiation from naive T cells (79), and IL-6 could also induce the generation of cytotoxic T cells (80). In our study, the levels of IL-6 in immunized birds were also enhanced, suggesting that immunization with rEm1a preparations induced host adaptive immune responses.

Host cellular immunity plays a dominant role in resisting the replications of *Eimeria* spp. parasites (3, 81). Two signals elicited by major histocompatibility complex (MHC) II and costimulatory molecules are required to activate the CD4^+^ T lymphocytes (82, 83). In addition, APCs or CD4^+^ Th cells are essential to induce the activation of CD8^+^ T lymphocytes (84, 85). Activated T lymphocytes will experience two major processes, proliferation and differentiation (86). In the present research, a CCK-8 assay was employed to evaluate the impacts of rEm1a on the proliferation of splenic lymphocytes isolated from immunized chickens *in vivo*. Splenocytes separated from nanosphere-vaccinated groups displayed greater proliferation than unencapsulated rEm1a control, suggesting that rEm1a that is formulated as PLGA or CS nanospheres could significantly enhance the proliferation of splenic lymphocytes in immunized birds. After activation and proliferation, T lymphocytes are polarized to different Th subsets, which initiate various types of immune responses (87). Here, we collected spleens to determine the proportions of CD4^+^ and CD8^+^ T lymphocytes. The proportions of CD4^+^ and CD8^+^ T lymphocytes were notably enhanced in all nanosphere-immunized birds compared to rEm1a-immunized ones. In host immunity against *E. mitis*, CD4^+^ T lymphocytes can generate Th cytokines and function in the formation of memory CD8^+^ T lymphocytes, while CD8^+^ T lymphocytes serve as the effector cells and exert cytotoxic effects (67, 88). Herein, rEm1a entrapped in emulsions or nanospheres could remarkably induce CD4^+^ and CD8^+^ T-cell polarization *in vivo*, which may further confer cellular and humoral resistance to *E. mitis*.

For vaccine studies against chicken coccidiosis, multiple vaccine adjuvants have been employed to induce prolonged and strengthened host immunity (89). Entrapped in PLGA nanospheres, recombinant *E. tenella* TA4 antigens showed enhanced immunoprotection against *E. tenella* infections (46). Immunization with recombinant *E. acervulina* emulsified in a novel adjuvant containing Carbopol, dimethyl dioctadecyl ammonium bromide, cholesterol, and Quil A could improve weight gains and inhibit outputs of fecal oocysts in *E. acervulina*-challenged birds (90). Similar results were also reported by Rafiqi et al. (91) in chickens immunized with Montanide™ ISA 71 VG encapsulated with recombinant *E. tenella* SO7 proteins. However, there is a lack of vaccine studies that directly compare the effect of different adjuvants. To determine the best adjuvant among the four tested ones, growth performance, oocyst output, and antigen-induced protective immunity in different groups were evaluated. Among the four different rEm1a preparations, only vaccination with the Em1a-PLGA nanospheres resulted in relatively better growth performance than the rEm1a protein. As for *E. mitis* oocyst output, birds immunized with the Em1a-PLGA nanospheres had the lowest oocyst output in feces. In addition, all Em1a-loaded emulsions and nanospheres could enhance the immunogenicity of the rEm1a antigen, whereas Em1a-PLGA nanospheres appeared to be the best preparation for eliciting humoral and cellular immune responses in challenged birds. Collectively, the PLGA nanosphere is associated with optimal protection in this study, and further testing and comparison are needed under field conditions.

## Conclusion

In summary, we cloned and expressed an electron transport pathway-related protein of Em1a. The rEm1a protein was encapsulated in two emulsions and two nanospheres to generate different rEm1a preparations, and the protective efficacy of the rEm1a preparations was evaluated in *E. mitis*-challenged chickens. All rEm1a-loaded preparations could elicit cellular and humoral immunity against *E. mitis*. Among the four tested preparations, PLGA nanospheres loaded with the rEm1a antigen could significantly improve growth performance and reduce *E. mitis* oocyst output of challenged birds. Thus, the PLGA nanosphere is associated with optimal protection against *E. mitis* infections in this study. Although the Em1a-PLGA nanosphere preparation could offer encouraging levels of immune protection to challenged birds, full protection against *E. mitis* was still unavailable. Further investigation is warranted with an emphasis on the optimization of the immunization regimen. Collectively, we provide a new perspective for developing effective vaccines against chicken coccidiosis.

## Data Availability Statement

The original contributions presented in the study are included in the article/**Supplementary Material**. Further inquiries can be directed to the corresponding author.

## Ethics Statement

The animal trials were approved by the Animal Ethics Committee, Nanjing Agriculture University, Nanjing, China. Approval number: NJAU.No20211223196 (for chicken trials), NJAU.No20211013152 (for mouse trials).

## Author Contributions

LX and XL designed the research. LX, ZY, WZ, and KH conducted the research. LX and ZY analyzed the data. LX, ZY, and MA wrote the article. RY, XS, and ML participated in the revision of the article. All authors contributed to the data interpretation and approved the final version of the article.

## Funding

This research was funded by a Joint Research Project between the National Natural Science Foundation of China and the Pakistan Science Foundation (NSFCPSF) (31661143017).

## Conflict of Interest

The authors declare that the research was conducted in the absence of any commercial or financial relationships that could be construed as a potential conflict of interest.

## Publisher’s Note

All claims expressed in this article are solely those of the authors and do not necessarily represent those of their affiliated organizations, or those of the publisher, the editors and the reviewers. Any product that may be evaluated in this article, or claim that may be made by its manufacturer, is not guaranteed or endorsed by the publisher.
