# Supplementary material for: PLGA Nanospheres as Delivery Platforms for Eimeria mitis 1a Protein: A Novel Strategy to Improve Specific Immunity
Source: Front Immunol. 2022 May 26;13:901758. doi: 10.3389/fimmu.2022.901758 (PMC9178187; doi:10.3389/fimmu.2022.901758)
Supplement: Supplementary file 1 [file Table_1.docx]

**Table S1.** Primers used for the molecular identification.

| Species | Name | Sequence (5′ - 3′) | Amplified fragment length (bp) |
| --- | --- | --- | --- |
| *E. acervulina* | Ea-F | GGCTTGGATGATGTTTGCTG | 321 |
|  | Ea-R | CGAACGCAATAACACACGCT |  |
| *E. brunetti* | Eb-F | GATCAGTTTGAGCAAACCTTCG | 311 |
|  | Eb-R | TGGTCTTCCGTACGTCGGAT |  |
| *E. necatrix* | En-F | TACATCCCAATCTTTGAATCG | 384 |
|  | En-R | GGCATACTAGCTTCGAGCAAC |  |
| *E. tenella* | Et-F | AATTTAGTCCATCGCAACCCT | 279 |
|  | Et-R | CGAGCGCTCTGCATACGACA |  |
| *E. maxima* | Em-F | GTGGGACTGTGGTGATGGGG | 205 |
|  | Em-R | ACCAGCATGCGCTCACAACCC |  |
| *E. mitis* | Emit-F | TATTTCCTGTCGTCGTCTCGC | 327 |
|  | Emit-R | GTATGCAAGAGAGAATCGGGA |  |
| *E. praecox* | Ep-F | CATCGGAATGGCTTTTTGAAAGCG | 215 |
|  | Ep-R | GCATGCGCTAACACCTCCCCTT |  |
